# Crystal structure of chlorido­[1-(4-nitro­phen­yl)thio­urea-κ*S*]bis­(tri­phenyl­phosphane-κ*P*)silver(I)

**DOI:** 10.1107/S2056989017006405

**Published:** 2017-05-09

**Authors:** Arunpatcha Nimthong-Roldán, Paramee Sripa, Yupa Wattanakanjana

**Affiliations:** a Department of Chemistry, Boston University, Boston, Massachusetts 02215, University; b Department of Chemistry, Faculty of Science, Prince of Songkla University, Hat Yai, Songkhla 90112 , Thailand

**Keywords:** crystal structure, N—H⋯Cl hydrogen bonding, intra­molecular hydrogen bonding, inter­molecular hydrogen bonding

## Abstract

In the title compound, [AgCl(C_7_H_7_N_3_O_2_S)(C_18_H_15_P)_2_], the Ag^I^ ion is in a distorted tetra­hedral coordination environment formed by P atoms from two tri­phenyl­phosphane ligands, one terminal S atom from the 1-(4-nitro­phen­yl)thio­urea ligand and a chloride ion. In the crystal, bifurcated (N—H)2⋯Cl hydrogen bonds [with graph-set motif *R*_2_^1^(6)] connect complex mol­ecules, forming zigzag chains along [001]. These chains are linked *via* weak C—H⋯O hydrogen bonds, forming a two-dimensional network parallel to (100). An intra­molecular N—H⋯Cl hydrogen bond forming an *S*(6) ring is also observed.

## Abstract 2

Reaction of silver(I) chloride with 1-(4-nitro­phen­yl)thio­urea and tri­phenyl­phosphane ligands of 1:2:1 ratio leads to the mononuclear complex [AgCl(C_7_H_7_N_3_O_2_S)(C_18_H_15_P)_2_]. In the crystal, bifurcated N—H⋯Cl and a weak C—H⋯O hydrogen bonds link mol­ecules into a two-dimensional network.

## Chemical context   

Studies of thio­urea and thio­urea derivatives have recently attracted considerable attention because of their variety of biological properties such as increasing technologies for plasma membrane proteomics (Cordwell & Thingholm, 2010 ▸), anti­microbial and cytotoxic activity (Bielenica *et al.*, 2015 ▸) and significant anti­fungal and anti-viral activity of curative rates (Wu *et al.*, 2012 ▸). Silver(I) complexes containing tri­phenyl­phosphane as precursors have been studied extensively for the preparation of mixed ligands with thio­urea derivatives (Mekarat *et al.*, 2014 ▸; Wattanakanjana *et al.*, 2014 ▸). Recently, we reported a complex that was prepared by reacting copper(I) chloride containing tri­phenyl­phosphane and 1-(4-nitro­phen­yl)thio­urea ligands (Nimthong-Roldán *et al.*, 2017 ▸). Herein, we report the crystal structure of the compound formed using silver(I) instead of copper(I) under the same conditions, [AgCl(C_7_H_7_N_3_O_2_S)(C_18_H_15_P)_2_] (I).
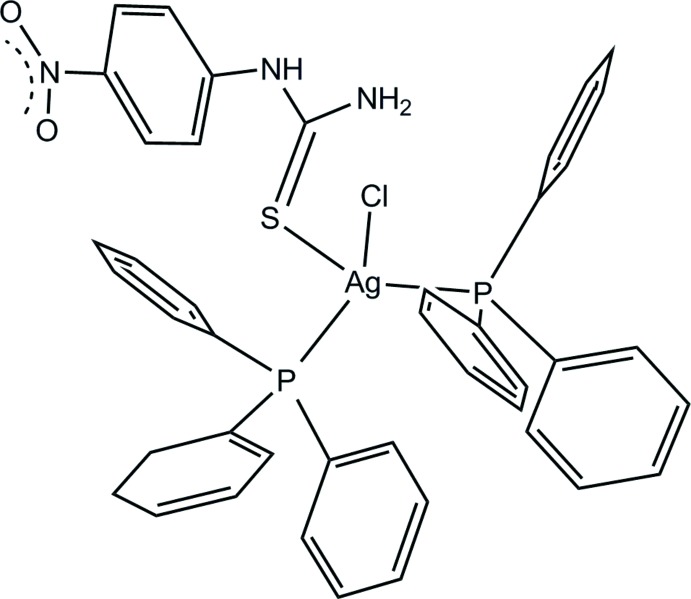


## Structural commentary   

**Figure 1:**
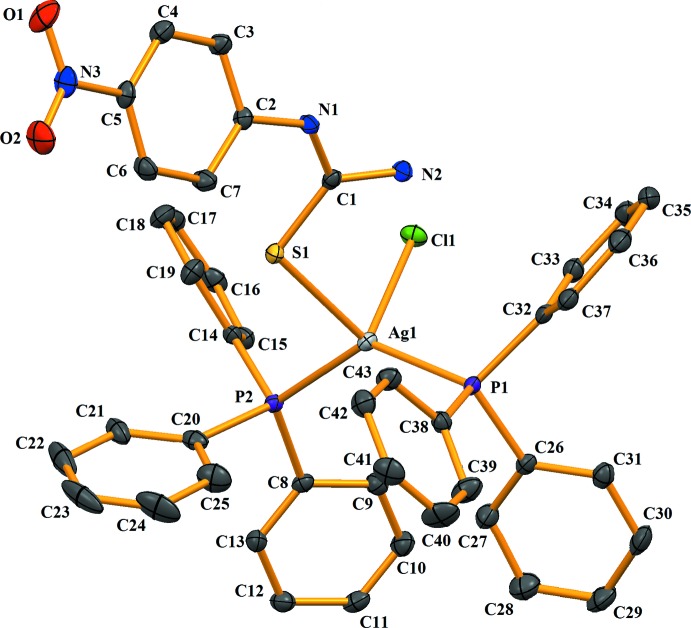
The mol­ecular structure of (I), with displacement ellipsoids drawn at the 50% probability level. All H atoms have been omitted for clarity.

In compound (I), tri­phenyl­phosphane, PPh_3_, and a 1-(4-nitro­phen­yl)thio­urea ligand, NPTU, as co-ligands coordinate the Ag^I^ ion with two P atoms from two PPh_3_ ligands, one terminal S atom from the NPTU ligand and one chloride ion, resulting in a distorted tetra­hedral environment (Fig. 1 ▸). The Ag—S bond length of 2.6316 (5) is similar to that of 2.603 (4) Å found in [Ag_2_Cl_2_(CH_5_N_3_S)_2_(C_18_H_15_P)_2_], (Wattanakanjana *et al.*, 2012 ▸). An intra­molecular N2—H2*B*⋯Cl1 hydrogen bond with graph-set motif *S*(6) is observed (Table 1 ▸).

## Supra­molecular features   

**Figure 2:**
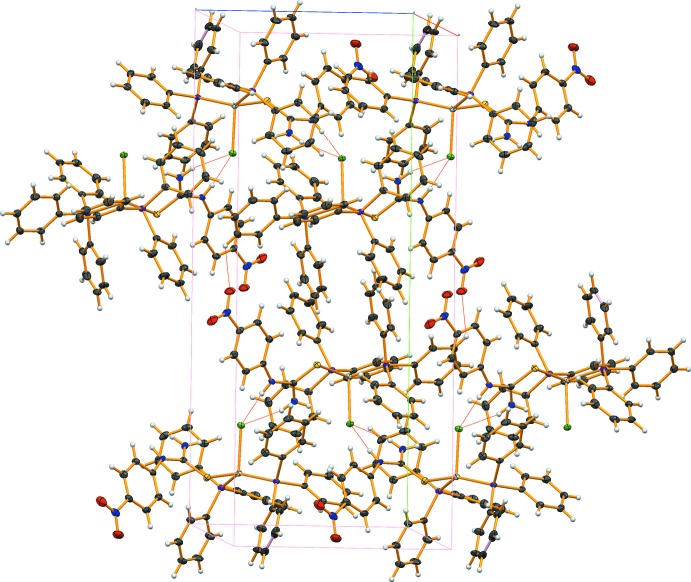
Part of the crystal structure of (I), showing the two-dimensional network formed by inter­molecular N—H⋯Cl and C—H⋯O hydrogen bonds (shown as dashed lines) parallel to (100).

In the crystal, N2—H2*A*⋯Cl1 and N1—H1⋯Cl1 hydrogen bonds link the mol­ecules, forming a zigzag chain along [001]. These chains are linked by weak C12—H12⋯O2 hydrogen bonds, leading to the formation of a two-dimensional network parallel to (100) (Fig. 2 ▸ and Table 1 ▸).

## Database survey   

A search of the Cambridge Structural Database (Version 5.37, Feb 2016 with two updates; Groom *et al.*, 2016 ▸) revealed no complexes with the 1-(4-nitro­phen­yl)thio­urea ligand, and only the crystal structure of the ligand itself has been reported (LONSEN; Xian *et al.*, 2008 ▸). A search for phenyl­thio­urea ligands with substitutions on the phenyl ring yielded 34 hits. Of these, four hits were Ag^I^ complexes, namely TUYZAQ (Wattanakanjana *et al.*, 2015 ▸), SUFDUU (Nimthong-Roldán *et al.*, 2015*b* ▸), WUFBIK (Nimthong-Roldán *et al.*, 2015*a* ▸), and XOFDED (Mekarat *et al.*, 2014 ▸)

## Synthesis and crystallization   

Tri­phenyl­phosphane, PPh_3_ (0.16 g, 0.51 mmol) was dissolved in 30 ml of aceto­nitrile at 340 K and then silver(I) chloride, AgCl (0.04 g, 0.25 mmol) , was added. The mixture was stirred for 3 h and then 1-(4-nitro­phen­yl)-2- thio­urea, NPTU (0.05 g, 0.25 mmol), was added. The resulting reaction mixture was heated under reflux for 3 h during which the precipitate gradually disappeared. The resulting clear solution was filtered and left to evaporate at room temperature. The crystalline complex, which deposited upon standing for a couple of days, was filtered off and dried *in vacuo* (0.16 g, 66% yield). M.p. 465–467 K. IR bands (KBr, cm^−1^): 3259 (*w*), 3134 (*w*), 3051(*w*), 2366 (*w*), 2345 (*w*), 1584 (*w*), 1509 (*w*), 1498 (*w*), 1458 (*w*), 1433 (*w*), 1399 (*w*), 1334 (*s*), 1297 (*w*), 1259 (*w*), 1181 (*w*), 1157 (*w*), 1110 (*w*), 1095 (*w*), 1027 (*w*), 998 (*w*), 890 (*w*), 851 (*w*), 746 (*m*), 720 (*w*), 694 (*s*), 670 (*w*), 594 (*w*), 515 (*m*), 501 (*m*), 491 (*m*).

## Refinement   

Crystal data, data collection and structure refinement details are summarized in Table 2 ▸. All H atoms attached to carbon atoms and atom H1 attached to nitro­gen atom N1 were positioned geometrically and constrained to ride on their parent atoms, with C—H = 0.95 Å and N1—H1 = 0.88 Å. The other nitro­gen-bound H atoms were located in difference-Fourier maps and were refined with an N—H distance restraint of 0.88 (2) Å. *U*_iso_(H) values were set to 1.2*U*_eq_(C/N). Reflections 1 1 0, 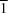 1 1, 0 2 0, 1 2 0, 0 4 0, 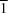 2 1, 0 2 1, 0 1 1, 1 0 0, −5 11, 13 8 1, 6 15 10, 12 10 4, 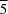 15 13, 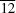 20 2, 0 22 11, 12 1 5, 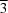 23 11, 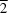 26 10, 4 9 12, 10 14 6, 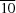 20 9, 7 22 7, 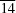 8 5, 10 10 7, 0 5 14, 7 5 10, 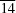 8 4, 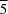 25 10, 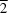 20 12 and 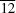 14 9 were affected by the beam stop and were omitted from the refinement.

## Supplementary Material

Crystal structure: contains datablock(s) I, global. DOI: 10.1107/S2056989017006405/lh5841sup1.cif

Structure factors: contains datablock(s) I. DOI: 10.1107/S2056989017006405/lh5841Isup2.hkl

CCDC reference: 1546767

Additional supporting information:  crystallographic information; 3D view; checkCIF report

## Figures and Tables

**Table 1:** Hydrogen-bond geometry (Å, °)

*D*—H⋯*A*	*D*—H	H⋯*A*	*D*⋯*A*	*D*—H⋯*A*
N1—H1⋯Cl1^i^	0.88	2.41	3.2454 (17)	159
N2—H2*A*⋯Cl1^i^	0.88 (2)	2.39 (2)	3.2257 (18)	160 (2)
N2—H2*B*⋯Cl1	0.87 (2)	2.50 (2)	3.3247 (18)	159 (2)
C12—H12⋯O2^ii^	0.95	2.60	3.272 (3)	129

Symmetry codes: (i) 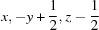; (ii) 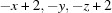.

**Table 2:** Experimental details

Crystal data	
Chemical formula	[AgCl(C_7_H_7_N_3_O_2_S)(C_18_H_15_P)_2_]
*M*_r_	865.07
Crystal system, space group	Monoclinic, *P*2_1_/*c*
Temperature (K)	100
*a*, *b*, *c* (Å)	11.8581 (2), 28.5087 (4), 12.0272 (2)
β (°)	104.9338 (17)
*V* (Å^3^)	3928.57 (11)
*Z*	4
Radiation type	Cu *K*α
μ (mm^−1^)	6.33
Crystal size (mm)	0.25 × 0.23 × 0.18

Data collection	
Diffractometer	Rigaku RAPID II curved image plate diffractometer
Absorption correction	Multi-scan (*SCALEPACK*; Otwinowski & Minor, 1997 ▸)
*T*_min_, *T*_max_	0.253, 0.395
No. of measured, independent and observed [*I* > 2σ(*I*)] reflections	73254, 7590, 7495
*R*_int_	0.051
(sin θ/λ)_max_ (Å^−1^)	0.618

Refinement	
*R*[*F* ^2^ > 2σ(*F* ^2^)], *wR*(*F* ^2^), *S*	0.027, 0.068, 1.08
No. of reflections	7590
No. of parameters	485
No. of restraints	2
H-atom treatment	H atoms treated by a mixture of independent and constrained refinement
Δρ_max_, Δρ_min_ (e Å^−3^)	0.43, −0.37

Computer programs: *CrystalClear-SM Expert* (Rigaku, 2014 ▸), *HKL-3000* (Otwinowski & Minor, 1997 ▸), *SHELXS97* and *SHELXL97* (Sheldrick, 2008 ▸), *SHELXL2014* (Sheldrick, 2015 ▸), *SHELXLE* (Hübschle *et al.*, 2011 ▸), *Mercury* (Macrae *et al.*, 2008 ▸) and *publCIF* (Westrip, 2010 ▸).

## supplementary crystallographic information

### Crystal data

**Table:**

[AgCl(C_7_H_7_N_3_O_2_S)(C_18_H_15_P)_2_]	*F*(000) = 1768
*M**_r_* = 865.07	*D*_x_ = 1.463 Mg m^−^^3^
Monoclinic, *P*2_1_/*c*	Cu *K*α radiation, λ = 1.54178 Å
*a* = 11.8581 (2) Å	Cell parameters from 73254 reflections
*b* = 28.5087 (4) Å	θ = 6.0–72.3°
*c* = 12.0272 (2) Å	µ = 6.33 mm^−^^1^
β = 104.9338 (17)°	*T* = 100 K
*V* = 3928.57 (11) Å^3^	Fragment, colourless
*Z* = 4	0.25 × 0.23 × 0.18 mm

### Data collection

**Table:**

Rigaku RAPID II curved image plate diffractometer	7590 independent reflections
Radiation source: microfocus X-ray tube	7495 reflections with *I* > 2σ(*I*)
Laterally graded multilayer (Goebel) mirror monochromator	*R*_int_ = 0.051
ω scans	θ_max_ = 72.3°, θ_min_ = 6.0°
Absorption correction: multi-scan (SCALEPACK; Otwinowski & Minor, 1997)	*h* = −13→14
*T*_min_ = 0.253, *T*_max_ = 0.395	*k* = −34→34
73254 measured reflections	*l* = −14→14

### Refinement

**Table:**

Refinement on *F*^2^	Secondary atom site location: difference Fourier map
Least-squares matrix: full	Hydrogen site location: mixed
*R*[*F*^2^ > 2σ(*F*^2^)] = 0.027	H atoms treated by a mixture of independent and constrained refinement
*wR*(*F*^2^) = 0.068	*w* = 1/[σ^2^(*F*_o_^2^) + (0.0286*P*)^2^ + 4.5052*P*] where *P* = (*F*_o_^2^ + 2*F*_c_^2^)/3
*S* = 1.08	(Δ/σ)_max_ = 0.002
7590 reflections	Δρ_max_ = 0.43 e Å^−^^3^
485 parameters	Δρ_min_ = −0.37 e Å^−^^3^
2 restraints	Extinction correction: SHELXL2014 (Sheldrick, 2015), Fc^*^=kFc[1+0.001xFc^2^λ^3^/sin(2θ)]^-1/4^
Primary atom site location: structure-invariant direct methods	Extinction coefficient: 0.00032 (3)

### Special details

**Table:**

Geometry. All esds (except the esd in the dihedral angle between two l.s. planes) are estimated using the full covariance matrix. The cell esds are taken into account individually in the estimation of esds in distances, angles and torsion angles; correlations between esds in cell parameters are only used when they are defined by crystal symmetry. An approximate (isotropic) treatment of cell esds is used for estimating esds involving l.s. planes.
Refinement. NH2 hydrogen positions were refined with an N-H distance restraint of 0.88 (2) Angstrom.

### Fractional atomic coordinates and isotropic or equivalent isotropic displacement parameters (Å^2^)

**Table:**

	*x*	*y*	*z*	*U*_iso_*/*U*_eq_	
C1	0.82775 (17)	0.17354 (7)	0.72805 (16)	0.0133 (4)	
C2	0.96312 (17)	0.14219 (7)	0.61654 (16)	0.0139 (4)	
C3	1.03535 (18)	0.16067 (7)	0.55233 (18)	0.0165 (4)	
H3	1.0332	0.1933	0.5361	0.020*	
C4	1.10993 (18)	0.13194 (7)	0.51217 (18)	0.0190 (4)	
H4	1.1578	0.1444	0.4670	0.023*	
C5	1.11381 (18)	0.08464 (7)	0.53871 (17)	0.0174 (4)	
C6	1.04603 (19)	0.06567 (7)	0.60585 (18)	0.0194 (4)	
H6	1.0514	0.0333	0.6249	0.023*	
C7	0.97043 (19)	0.09454 (7)	0.64472 (18)	0.0187 (4)	
H7	0.9235	0.0819	0.6906	0.022*	
C8	0.74747 (17)	0.13044 (7)	1.26846 (16)	0.0138 (4)	
C9	0.65144 (19)	0.15917 (7)	1.26283 (18)	0.0179 (4)	
H9	0.6270	0.1806	1.2007	0.022*	
C10	0.5909 (2)	0.15680 (7)	1.3476 (2)	0.0210 (5)	
H10	0.5264	0.1770	1.3442	0.025*	
C11	0.62485 (18)	0.12492 (7)	1.43677 (18)	0.0189 (4)	
H11	0.5831	0.1230	1.4943	0.023*	
C12	0.71971 (18)	0.09583 (7)	1.44220 (17)	0.0190 (4)	
H12	0.7422	0.0737	1.5030	0.023*	
C13	0.78211 (18)	0.09879 (7)	1.35929 (17)	0.0166 (4)	
H13	0.8482	0.0793	1.3644	0.020*	
C14	0.93770 (17)	0.17768 (6)	1.20401 (16)	0.0136 (4)	
C15	0.93999 (18)	0.20815 (7)	1.29504 (17)	0.0164 (4)	
H15	0.8819	0.2059	1.3364	0.020*	
C16	1.02734 (19)	0.24204 (7)	1.32554 (18)	0.0205 (4)	
H16	1.0283	0.2629	1.3873	0.025*	
C17	1.11251 (19)	0.24539 (7)	1.26628 (19)	0.0225 (5)	
H17	1.1731	0.2680	1.2886	0.027*	
C18	1.10938 (19)	0.21572 (8)	1.17397 (19)	0.0235 (5)	
H18	1.1675	0.2182	1.1326	0.028*	
C19	1.02183 (18)	0.18265 (8)	1.14223 (18)	0.0192 (4)	
H19	1.0188	0.1631	1.0776	0.023*	
C20	0.89340 (19)	0.07855 (7)	1.15416 (17)	0.0181 (4)	
C21	1.0099 (2)	0.06968 (8)	1.21129 (18)	0.0233 (5)	
H21	1.0565	0.0936	1.2558	0.028*	
C22	1.0570 (3)	0.02535 (9)	1.2023 (2)	0.0361 (6)	
H22	1.1365	0.0193	1.2399	0.043*	
C23	0.9895 (3)	−0.00974 (9)	1.1395 (2)	0.0416 (7)	
H23	1.0225	−0.0397	1.1336	0.050*	
C24	0.8739 (3)	−0.00123 (8)	1.0852 (2)	0.0399 (7)	
H24	0.8268	−0.0256	1.0435	0.048*	
C25	0.8263 (2)	0.04271 (8)	1.0911 (2)	0.0294 (5)	
H25	0.7472	0.0485	1.0518	0.035*	
C26	0.40317 (17)	0.12920 (7)	0.91747 (17)	0.0144 (4)	
C27	0.43678 (19)	0.11373 (8)	1.03101 (18)	0.0211 (4)	
H27	0.5168	0.1076	1.0661	0.025*	
C28	0.3548 (2)	0.10713 (8)	1.0934 (2)	0.0258 (5)	
H28	0.3784	0.0962	1.1705	0.031*	
C29	0.2380 (2)	0.11659 (8)	1.0429 (2)	0.0251 (5)	
H29	0.1817	0.1122	1.0855	0.030*	
C30	0.20359 (19)	0.13244 (8)	0.9305 (2)	0.0236 (5)	
H30	0.1237	0.1393	0.8966	0.028*	
C31	0.28542 (18)	0.13847 (7)	0.86665 (19)	0.0189 (4)	
H31	0.2613	0.1488	0.7891	0.023*	
C32	0.45452 (17)	0.17405 (7)	0.72094 (17)	0.0145 (4)	
C33	0.45617 (18)	0.22199 (7)	0.74552 (19)	0.0190 (4)	
H33	0.4862	0.2326	0.8224	0.023*	
C34	0.41417 (19)	0.25422 (7)	0.6582 (2)	0.0229 (5)	
H34	0.4137	0.2867	0.6758	0.028*	
C35	0.37279 (19)	0.23917 (8)	0.5452 (2)	0.0237 (5)	
H35	0.3453	0.2613	0.4853	0.028*	
C36	0.37170 (19)	0.19174 (8)	0.52005 (19)	0.0225 (4)	
H36	0.3438	0.1814	0.4427	0.027*	
C37	0.41126 (18)	0.15910 (7)	0.60767 (18)	0.0178 (4)	
H37	0.4087	0.1266	0.5901	0.021*	
C38	0.51237 (17)	0.07648 (7)	0.77110 (17)	0.0151 (4)	
C39	0.4272 (2)	0.04295 (8)	0.7711 (2)	0.0248 (5)	
H39	0.3669	0.0495	0.8077	0.030*	
C40	0.4301 (2)	−0.00014 (8)	0.7180 (2)	0.0308 (5)	
H40	0.3719	−0.0229	0.7187	0.037*	
C41	0.5173 (2)	−0.01007 (7)	0.66374 (19)	0.0252 (5)	
H41	0.5186	−0.0395	0.6270	0.030*	
C42	0.60271 (19)	0.02319 (7)	0.66335 (18)	0.0212 (4)	
H42	0.6624	0.0166	0.6259	0.025*	
C43	0.60110 (19)	0.06598 (7)	0.71745 (18)	0.0186 (4)	
H43	0.6606	0.0884	0.7182	0.022*	
N1	0.88013 (15)	0.17306 (6)	0.64028 (14)	0.0148 (3)	
H1	0.8585	0.1958	0.5899	0.018*	
N2	0.74178 (15)	0.20477 (6)	0.71595 (15)	0.0162 (3)	
H2A	0.727 (2)	0.2227 (8)	0.6547 (17)	0.019*	
H2B	0.721 (2)	0.2127 (8)	0.7774 (17)	0.019*	
N3	1.18951 (16)	0.05374 (6)	0.49253 (16)	0.0226 (4)	
O1	1.25615 (14)	0.07175 (6)	0.44157 (15)	0.0309 (4)	
O2	1.18095 (15)	0.01114 (6)	0.50467 (15)	0.0314 (4)	
S1	0.86821 (4)	0.13848 (2)	0.84576 (4)	0.01445 (10)	
Cl1	0.73410 (4)	0.24665 (2)	0.97189 (4)	0.01787 (10)	
Ag1	0.70884 (2)	0.15453 (2)	0.95816 (2)	0.01254 (6)	
P1	0.51614 (4)	0.13383 (2)	0.83979 (4)	0.01185 (10)	
P2	0.82115 (4)	0.13508 (2)	1.15318 (4)	0.01158 (10)	

### Atomic displacement parameters (Å^2^)

**Table:**

	*U*^11^	*U*^22^	*U*^33^	*U*^12^	*U*^13^	*U*^23^
C1	0.0103 (9)	0.0120 (9)	0.0179 (9)	−0.0021 (7)	0.0045 (7)	−0.0016 (7)
C2	0.0143 (10)	0.0136 (9)	0.0140 (9)	0.0017 (7)	0.0042 (7)	−0.0015 (7)
C3	0.0171 (11)	0.0123 (9)	0.0214 (10)	−0.0012 (7)	0.0072 (8)	−0.0011 (7)
C4	0.0163 (11)	0.0192 (10)	0.0233 (10)	−0.0021 (8)	0.0082 (8)	−0.0030 (8)
C5	0.0146 (10)	0.0176 (10)	0.0202 (10)	0.0040 (8)	0.0049 (8)	−0.0038 (8)
C6	0.0235 (11)	0.0126 (9)	0.0230 (10)	0.0048 (8)	0.0076 (9)	0.0022 (8)
C7	0.0222 (11)	0.0156 (10)	0.0214 (10)	0.0022 (8)	0.0113 (8)	0.0022 (8)
C8	0.0136 (10)	0.0127 (9)	0.0157 (9)	−0.0030 (7)	0.0047 (7)	−0.0008 (7)
C9	0.0188 (11)	0.0146 (10)	0.0213 (10)	0.0012 (8)	0.0068 (8)	0.0018 (8)
C10	0.0192 (12)	0.0178 (10)	0.0293 (12)	0.0029 (8)	0.0118 (9)	−0.0001 (8)
C11	0.0185 (11)	0.0180 (10)	0.0234 (10)	−0.0045 (8)	0.0113 (8)	−0.0033 (8)
C12	0.0207 (11)	0.0178 (10)	0.0192 (10)	−0.0020 (8)	0.0064 (8)	0.0031 (8)
C13	0.0140 (10)	0.0152 (9)	0.0208 (10)	0.0011 (7)	0.0052 (8)	0.0019 (8)
C14	0.0120 (10)	0.0103 (9)	0.0179 (9)	0.0008 (7)	0.0028 (7)	0.0028 (7)
C15	0.0173 (10)	0.0130 (9)	0.0192 (10)	0.0013 (7)	0.0056 (8)	0.0005 (7)
C16	0.0251 (12)	0.0116 (9)	0.0219 (10)	−0.0014 (8)	0.0009 (9)	−0.0014 (8)
C17	0.0177 (11)	0.0165 (10)	0.0289 (11)	−0.0052 (8)	−0.0020 (9)	0.0039 (8)
C18	0.0150 (11)	0.0290 (12)	0.0278 (11)	−0.0047 (9)	0.0077 (9)	0.0037 (9)
C19	0.0136 (10)	0.0227 (11)	0.0215 (10)	−0.0018 (8)	0.0050 (8)	−0.0021 (8)
C20	0.0256 (11)	0.0145 (10)	0.0177 (9)	0.0016 (8)	0.0116 (8)	0.0019 (8)
C21	0.0290 (12)	0.0211 (11)	0.0218 (10)	0.0117 (9)	0.0105 (9)	0.0064 (8)
C22	0.0482 (16)	0.0373 (14)	0.0283 (12)	0.0303 (12)	0.0197 (11)	0.0134 (11)
C23	0.085 (2)	0.0183 (12)	0.0312 (13)	0.0258 (13)	0.0327 (14)	0.0094 (10)
C24	0.076 (2)	0.0108 (11)	0.0357 (14)	0.0014 (12)	0.0192 (14)	−0.0020 (9)
C25	0.0399 (15)	0.0176 (11)	0.0316 (12)	−0.0019 (10)	0.0110 (11)	−0.0044 (9)
C26	0.0130 (10)	0.0102 (9)	0.0215 (10)	−0.0009 (7)	0.0072 (8)	−0.0034 (7)
C27	0.0159 (11)	0.0244 (11)	0.0234 (10)	0.0003 (8)	0.0061 (8)	0.0003 (8)
C28	0.0268 (12)	0.0283 (12)	0.0262 (11)	−0.0029 (9)	0.0137 (9)	0.0009 (9)
C29	0.0224 (12)	0.0209 (11)	0.0390 (13)	−0.0035 (9)	0.0208 (10)	−0.0038 (9)
C30	0.0125 (11)	0.0210 (11)	0.0400 (13)	−0.0005 (8)	0.0115 (9)	−0.0033 (9)
C31	0.0152 (10)	0.0157 (10)	0.0255 (10)	0.0003 (8)	0.0049 (8)	−0.0028 (8)
C32	0.0082 (9)	0.0146 (9)	0.0218 (10)	−0.0002 (7)	0.0056 (7)	0.0013 (7)
C33	0.0131 (10)	0.0168 (10)	0.0262 (10)	0.0000 (8)	0.0034 (8)	0.0000 (8)
C34	0.0152 (11)	0.0150 (10)	0.0380 (12)	0.0000 (8)	0.0059 (9)	0.0027 (9)
C35	0.0147 (11)	0.0254 (11)	0.0299 (11)	0.0012 (8)	0.0041 (9)	0.0124 (9)
C36	0.0159 (11)	0.0321 (12)	0.0201 (10)	−0.0002 (9)	0.0054 (8)	0.0043 (9)
C37	0.0121 (10)	0.0200 (10)	0.0224 (10)	−0.0012 (7)	0.0064 (8)	−0.0004 (8)
C38	0.0147 (10)	0.0120 (9)	0.0177 (9)	0.0017 (7)	0.0027 (8)	0.0005 (7)
C39	0.0241 (12)	0.0201 (11)	0.0333 (12)	−0.0063 (9)	0.0131 (10)	−0.0074 (9)
C40	0.0374 (14)	0.0161 (11)	0.0423 (14)	−0.0132 (10)	0.0165 (11)	−0.0091 (10)
C41	0.0345 (13)	0.0134 (10)	0.0283 (11)	−0.0011 (9)	0.0089 (10)	−0.0050 (8)
C42	0.0228 (11)	0.0169 (10)	0.0252 (10)	0.0034 (8)	0.0088 (9)	−0.0027 (8)
C43	0.0185 (11)	0.0142 (10)	0.0242 (10)	−0.0020 (8)	0.0073 (8)	−0.0027 (8)
N1	0.0176 (9)	0.0113 (8)	0.0181 (8)	0.0044 (6)	0.0091 (7)	0.0038 (6)
N2	0.0171 (9)	0.0159 (8)	0.0181 (8)	0.0049 (6)	0.0090 (7)	0.0037 (7)
N3	0.0205 (10)	0.0211 (9)	0.0262 (9)	0.0052 (7)	0.0063 (8)	−0.0058 (7)
O1	0.0248 (9)	0.0320 (9)	0.0425 (10)	0.0001 (7)	0.0205 (8)	−0.0101 (7)
O2	0.0366 (10)	0.0196 (8)	0.0410 (10)	0.0112 (7)	0.0158 (8)	−0.0010 (7)
S1	0.0120 (2)	0.0160 (2)	0.0164 (2)	0.00335 (17)	0.00569 (17)	0.00330 (17)
Cl1	0.0236 (3)	0.0121 (2)	0.0184 (2)	−0.00173 (17)	0.00615 (18)	−0.00192 (16)
Ag1	0.00999 (9)	0.01314 (8)	0.01461 (8)	−0.00147 (4)	0.00338 (6)	0.00063 (5)
P1	0.0085 (2)	0.0110 (2)	0.0165 (2)	−0.00051 (16)	0.00401 (18)	−0.00144 (17)
P2	0.0108 (2)	0.0098 (2)	0.0145 (2)	−0.00040 (17)	0.00404 (18)	0.00016 (17)

### Geometric parameters (Å, º)

**Table:**

C1—N2	1.333 (3)	C24—C25	1.384 (3)
C1—N1	1.356 (2)	C24—H24	0.9500
C1—S1	1.698 (2)	C25—H25	0.9500
C2—C3	1.396 (3)	C26—C27	1.392 (3)
C2—C7	1.397 (3)	C26—C31	1.398 (3)
C2—N1	1.403 (2)	C26—P1	1.825 (2)
C3—C4	1.381 (3)	C27—C28	1.386 (3)
C3—H3	0.9500	C27—H27	0.9500
C4—C5	1.384 (3)	C28—C29	1.387 (3)
C4—H4	0.9500	C28—H28	0.9500
C5—C6	1.388 (3)	C29—C30	1.384 (3)
C5—N3	1.465 (3)	C29—H29	0.9500
C6—C7	1.384 (3)	C30—C31	1.394 (3)
C6—H6	0.9500	C30—H30	0.9500
C7—H7	0.9500	C31—H31	0.9500
C8—C9	1.390 (3)	C32—C37	1.393 (3)
C8—C13	1.395 (3)	C32—C33	1.398 (3)
C8—P2	1.824 (2)	C32—P1	1.831 (2)
C9—C10	1.391 (3)	C33—C34	1.388 (3)
C9—H9	0.9500	C33—H33	0.9500
C10—C11	1.385 (3)	C34—C35	1.389 (3)
C10—H10	0.9500	C34—H34	0.9500
C11—C12	1.385 (3)	C35—C36	1.385 (3)
C11—H11	0.9500	C35—H35	0.9500
C12—C13	1.389 (3)	C36—C37	1.393 (3)
C12—H12	0.9500	C36—H36	0.9500
C13—H13	0.9500	C37—H37	0.9500
C14—C15	1.392 (3)	C38—C39	1.391 (3)
C14—C19	1.396 (3)	C38—C43	1.401 (3)
C14—P2	1.823 (2)	C38—P1	1.827 (2)
C15—C16	1.395 (3)	C39—C40	1.389 (3)
C15—H15	0.9500	C39—H39	0.9500
C16—C17	1.381 (3)	C40—C41	1.387 (3)
C16—H16	0.9500	C40—H40	0.9500
C17—C18	1.389 (3)	C41—C42	1.388 (3)
C17—H17	0.9500	C41—H41	0.9500
C18—C19	1.381 (3)	C42—C43	1.385 (3)
C18—H18	0.9500	C42—H42	0.9500
C19—H19	0.9500	C43—H43	0.9500
C20—C25	1.394 (3)	N1—H1	0.8800
C20—C21	1.398 (3)	N2—H2A	0.877 (16)
C20—P2	1.824 (2)	N2—H2B	0.870 (16)
C21—C22	1.397 (3)	N3—O1	1.230 (3)
C21—H21	0.9500	N3—O2	1.231 (2)
C22—C23	1.379 (4)	S1—Ag1	2.6316 (5)
C22—H22	0.9500	Cl1—Ag1	2.6435 (5)
C23—C24	1.379 (4)	Ag1—P1	2.4330 (5)
C23—H23	0.9500	Ag1—P2	2.4440 (5)
			
N2—C1—N1	114.37 (17)	C28—C27—H27	119.6
N2—C1—S1	121.88 (15)	C26—C27—H27	119.6
N1—C1—S1	123.74 (15)	C27—C28—C29	119.8 (2)
C3—C2—C7	119.50 (18)	C27—C28—H28	120.1
C3—C2—N1	116.00 (17)	C29—C28—H28	120.1
C7—C2—N1	124.28 (18)	C30—C29—C28	120.1 (2)
C4—C3—C2	120.63 (19)	C30—C29—H29	120.0
C4—C3—H3	119.7	C28—C29—H29	120.0
C2—C3—H3	119.7	C29—C30—C31	120.4 (2)
C3—C4—C5	118.92 (19)	C29—C30—H30	119.8
C3—C4—H4	120.5	C31—C30—H30	119.8
C5—C4—H4	120.5	C30—C31—C26	119.6 (2)
C4—C5—C6	121.62 (19)	C30—C31—H31	120.2
C4—C5—N3	119.05 (19)	C26—C31—H31	120.2
C6—C5—N3	119.31 (18)	C37—C32—C33	119.18 (19)
C7—C6—C5	119.18 (19)	C37—C32—P1	122.97 (15)
C7—C6—H6	120.4	C33—C32—P1	117.79 (15)
C5—C6—H6	120.4	C34—C33—C32	120.3 (2)
C6—C7—C2	120.08 (19)	C34—C33—H33	119.8
C6—C7—H7	120.0	C32—C33—H33	119.8
C2—C7—H7	120.0	C33—C34—C35	120.2 (2)
C9—C8—C13	119.44 (18)	C33—C34—H34	119.9
C9—C8—P2	117.84 (15)	C35—C34—H34	119.9
C13—C8—P2	122.70 (15)	C36—C35—C34	119.7 (2)
C8—C9—C10	120.44 (19)	C36—C35—H35	120.1
C8—C9—H9	119.8	C34—C35—H35	120.1
C10—C9—H9	119.8	C35—C36—C37	120.3 (2)
C11—C10—C9	119.8 (2)	C35—C36—H36	119.8
C11—C10—H10	120.1	C37—C36—H36	119.8
C9—C10—H10	120.1	C32—C37—C36	120.2 (2)
C10—C11—C12	120.00 (19)	C32—C37—H37	119.9
C10—C11—H11	120.0	C36—C37—H37	119.9
C12—C11—H11	120.0	C39—C38—C43	118.99 (18)
C11—C12—C13	120.40 (19)	C39—C38—P1	123.24 (16)
C11—C12—H12	119.8	C43—C38—P1	117.76 (15)
C13—C12—H12	119.8	C40—C39—C38	120.3 (2)
C12—C13—C8	119.86 (19)	C40—C39—H39	119.9
C12—C13—H13	120.1	C38—C39—H39	119.9
C8—C13—H13	120.1	C41—C40—C39	120.4 (2)
C15—C14—C19	118.90 (18)	C41—C40—H40	119.8
C15—C14—P2	122.60 (15)	C39—C40—H40	119.8
C19—C14—P2	118.24 (15)	C40—C41—C42	119.7 (2)
C14—C15—C16	120.11 (19)	C40—C41—H41	120.2
C14—C15—H15	119.9	C42—C41—H41	120.2
C16—C15—H15	119.9	C43—C42—C41	120.1 (2)
C17—C16—C15	120.3 (2)	C43—C42—H42	119.9
C17—C16—H16	119.9	C41—C42—H42	119.9
C15—C16—H16	119.9	C42—C43—C38	120.50 (19)
C16—C17—C18	119.86 (19)	C42—C43—H43	119.7
C16—C17—H17	120.1	C38—C43—H43	119.7
C18—C17—H17	120.1	C1—N1—C2	130.59 (17)
C19—C18—C17	120.0 (2)	C1—N1—H1	114.7
C19—C18—H18	120.0	C2—N1—H1	114.7
C17—C18—H18	120.0	C1—N2—H2A	117.7 (16)
C18—C19—C14	120.8 (2)	C1—N2—H2B	117.4 (16)
C18—C19—H19	119.6	H2A—N2—H2B	122 (2)
C14—C19—H19	119.6	O1—N3—O2	123.66 (18)
C25—C20—C21	119.2 (2)	O1—N3—C5	118.22 (18)
C25—C20—P2	116.29 (17)	O2—N3—C5	118.10 (18)
C21—C20—P2	124.46 (17)	C1—S1—Ag1	103.88 (7)
C22—C21—C20	119.3 (2)	P1—Ag1—P2	134.741 (17)
C22—C21—H21	120.3	P1—Ag1—S1	110.349 (16)
C20—C21—H21	120.3	P2—Ag1—S1	99.654 (16)
C23—C22—C21	120.8 (3)	P1—Ag1—Cl1	110.591 (16)
C23—C22—H22	119.6	P2—Ag1—Cl1	98.075 (15)
C21—C22—H22	119.6	S1—Ag1—Cl1	96.894 (15)
C22—C23—C24	119.8 (2)	C26—P1—C38	103.36 (9)
C22—C23—H23	120.1	C26—P1—C32	104.31 (9)
C24—C23—H23	120.1	C38—P1—C32	104.41 (9)
C23—C24—C25	120.3 (3)	C26—P1—Ag1	114.88 (7)
C23—C24—H24	119.9	C38—P1—Ag1	113.04 (7)
C25—C24—H24	119.9	C32—P1—Ag1	115.51 (6)
C24—C25—C20	120.5 (3)	C14—P2—C20	105.80 (9)
C24—C25—H25	119.7	C14—P2—C8	105.29 (9)
C20—C25—H25	119.7	C20—P2—C8	104.31 (9)
C27—C26—C31	119.35 (19)	C14—P2—Ag1	110.44 (6)
C27—C26—P1	117.57 (15)	C20—P2—Ag1	110.21 (7)
C31—C26—P1	123.02 (16)	C8—P2—Ag1	119.78 (7)
C28—C27—C26	120.7 (2)		
			
C7—C2—C3—C4	2.9 (3)	C38—C39—C40—C41	−0.4 (4)
N1—C2—C3—C4	−171.96 (18)	C39—C40—C41—C42	0.4 (4)
C2—C3—C4—C5	−1.4 (3)	C40—C41—C42—C43	0.4 (3)
C3—C4—C5—C6	−1.0 (3)	C41—C42—C43—C38	−1.2 (3)
C3—C4—C5—N3	177.66 (18)	C39—C38—C43—C42	1.2 (3)
C4—C5—C6—C7	1.7 (3)	P1—C38—C43—C42	−179.65 (16)
N3—C5—C6—C7	−176.90 (19)	N2—C1—N1—C2	−171.97 (19)
C5—C6—C7—C2	−0.1 (3)	S1—C1—N1—C2	9.2 (3)
C3—C2—C7—C6	−2.1 (3)	C3—C2—N1—C1	−153.6 (2)
N1—C2—C7—C6	172.30 (19)	C7—C2—N1—C1	31.8 (3)
C13—C8—C9—C10	0.6 (3)	C4—C5—N3—O1	7.4 (3)
P2—C8—C9—C10	179.24 (16)	C6—C5—N3—O1	−173.91 (19)
C8—C9—C10—C11	−1.4 (3)	C4—C5—N3—O2	−171.0 (2)
C9—C10—C11—C12	0.7 (3)	C6—C5—N3—O2	7.7 (3)
C10—C11—C12—C13	0.8 (3)	N2—C1—S1—Ag1	7.68 (17)
C11—C12—C13—C8	−1.5 (3)	N1—C1—S1—Ag1	−173.58 (15)
C9—C8—C13—C12	0.8 (3)	C27—C26—P1—C38	91.38 (17)
P2—C8—C13—C12	−177.71 (15)	C31—C26—P1—C38	−85.81 (18)
C19—C14—C15—C16	−1.9 (3)	C27—C26—P1—C32	−159.69 (16)
P2—C14—C15—C16	−175.92 (15)	C31—C26—P1—C32	23.13 (19)
C14—C15—C16—C17	−0.5 (3)	C27—C26—P1—Ag1	−32.24 (18)
C15—C16—C17—C18	1.7 (3)	C31—C26—P1—Ag1	150.58 (15)
C16—C17—C18—C19	−0.6 (3)	C39—C38—P1—C26	8.1 (2)
C17—C18—C19—C14	−1.8 (3)	C43—C38—P1—C26	−170.92 (16)
C15—C14—C19—C18	3.1 (3)	C39—C38—P1—C32	−100.71 (19)
P2—C14—C19—C18	177.33 (17)	C43—C38—P1—C32	80.22 (17)
C25—C20—C21—C22	1.1 (3)	C39—C38—P1—Ag1	132.96 (17)
P2—C20—C21—C22	−177.62 (17)	C43—C38—P1—Ag1	−46.11 (17)
C20—C21—C22—C23	−1.0 (3)	C37—C32—P1—C26	−104.98 (18)
C21—C22—C23—C24	−0.3 (4)	C33—C32—P1—C26	77.84 (17)
C22—C23—C24—C25	1.6 (4)	C37—C32—P1—C38	3.18 (19)
C23—C24—C25—C20	−1.6 (4)	C33—C32—P1—C38	−174.00 (16)
C21—C20—C25—C24	0.2 (3)	C37—C32—P1—Ag1	127.96 (16)
P2—C20—C25—C24	179.03 (19)	C33—C32—P1—Ag1	−49.22 (17)
C31—C26—C27—C28	0.5 (3)	C15—C14—P2—C20	−126.80 (16)
P1—C26—C27—C28	−176.77 (17)	C19—C14—P2—C20	59.16 (17)
C26—C27—C28—C29	−0.8 (3)	C15—C14—P2—C8	−16.70 (18)
C27—C28—C29—C30	0.2 (3)	C19—C14—P2—C8	169.26 (16)
C28—C29—C30—C31	0.8 (3)	C15—C14—P2—Ag1	113.94 (15)
C29—C30—C31—C26	−1.1 (3)	C19—C14—P2—Ag1	−60.09 (16)
C27—C26—C31—C30	0.5 (3)	C25—C20—P2—C14	−163.63 (17)
P1—C26—C31—C30	177.59 (16)	C21—C20—P2—C14	15.1 (2)
C37—C32—C33—C34	0.6 (3)	C25—C20—P2—C8	85.58 (18)
P1—C32—C33—C34	177.89 (16)	C21—C20—P2—C8	−95.71 (19)
C32—C33—C34—C35	−1.6 (3)	C25—C20—P2—Ag1	−44.22 (18)
C33—C34—C35—C36	1.1 (3)	C21—C20—P2—Ag1	134.49 (16)
C34—C35—C36—C37	0.4 (3)	C9—C8—P2—C14	92.78 (17)
C33—C32—C37—C36	0.9 (3)	C13—C8—P2—C14	−88.67 (17)
P1—C32—C37—C36	−176.27 (16)	C9—C8—P2—C20	−156.06 (16)
C35—C36—C37—C32	−1.4 (3)	C13—C8—P2—C20	22.50 (19)
C43—C38—C39—C40	−0.4 (3)	C9—C8—P2—Ag1	−32.22 (18)
P1—C38—C39—C40	−179.50 (19)	C13—C8—P2—Ag1	146.34 (14)

### Hydrogen-bond geometry (Å, º)

**Table:**

*D*—H···*A*	*D*—H	H···*A*	*D*···*A*	*D*—H···*A*
N1—H1···Cl1^i^	0.88	2.41	3.2454 (17)	159
N2—H2*A*···Cl1^i^	0.88 (2)	2.39 (2)	3.2257 (18)	160 (2)
N2—H2*B*···Cl1	0.87 (2)	2.50 (2)	3.3247 (18)	159 (2)
C12—H12···O2^ii^	0.95	2.60	3.272 (3)	129

Symmetry codes: (i) *x*, −*y*+1/2, *z*−1/2; (ii) −*x*+2, −*y*, −*z*+2.
